# Super Oxygenation, a Reply to Criticism and Strictures

**Published:** 1869-06-15

**Authors:** S. S. Wallihan

**Affiliations:** Chicago


### *Super Oxygenation, A Reply to Criticism and Strictures.*

BY S. S. WALLIHAN, M. D., CHICAGO.

Since several of your patrons have brought my previous paper on Super-oxygenation, into a somewhat questionable notoriety by a pointed denial and insinuation of intentional unfairness, in one instance, and by what might be termed a specie of *reductio ad absurdum*, in another, it seems incumbent on me to briefly set things to rights.

Your correspondent at Zanesville Ohio,—Dr. McElroy, in his attempt to analyze the results of the use of artificial oxygen and other oxygenizing agents, in accordance with his somewhat peculiar and not any too clearly stated theory of vital dynamics and the invariable unity of origin of all pathological conditions, applies the usually received and constantly repeated opinions concerning the therapeutic nature of these agents. Virtually admitting that he has had not the slightest experience with any form of artificial oxygen, he nevertheless reaches the conclusion that all its therapeutic effects may be arrived at through the time-honored means of the bromides, the ferrides and the quiniades. He makes the fatal but very common, and therefore probable excusable, mistake of assuming that a little more oxygen is nothing more nor less than a little more fresh air; and that it may be obtained just as effectually by a sojourn at the sea-shore, out-door labor, etc. My position, and that of every one who has fully investigated the therapeutic nature of oxygen and its gaseous compounds, is, that its *physiological* relations are definite and limited, while its nature and potency as a *remedial* or *therapeutic* agent are as yet very little known, entirely unlimited and as different from the former as though it were in reality another element. Throwing aside previous convictions,—the result of stereotyped chemical assumptions,—if Dr. McElroy will administer to a properly selected subject, even a few pints a day of artificial oxygen, duly diluted and modified,—which trifling quantity could be easily secured in its ordinary condition, from the atmosphere, by simply taking a few extra inspirations,—he will very readily convince himself that oxygen in one form is quite different from the same element in a slightly different form. Chemistry is full of even more striking anomalies of this very kind. Consider the innumerable compouuds of the three commonest elements, carbon, oxygen and hydrogén. The ultimate chemical composition of the most deadly vegetable poisons and of some of the blandest forms of food is almost identical. The contents of the rag pickers' basket, and the tempting sweets in the confectioner's window are chemically the same. But will we attempt to philosophize our digestive organs into an indiscriminate and indifferent use of sugar and cotton batting?

Dr. McElroy also assumes that all my patients,—at least all those reported as essentially benefited by a course of super-oxygenation,—belonged to " the better classes, " and were suffering from lack of wholesome exercise,—in short were *lazy*. This assumption was as far from the facts in the case as the former one respecting the therapeutic nature of oxygen &c. In at least four of the six cases given, the patients were of the medium class and if anything, *over-active* in their habits. The excessive and constant over-work of one in particular retarded his treatment materially. Plenty of fresh air and the sea-side are most excellent as hygienic, and in a degree, therapeutic considerations; but the limitless air of heaven and all the sea-shores from Greenland to the *Terra del Fuego* have failed to remove positive morbid conditions and change abnormal vital processes, as has been and can be accomplished by the agents in question, when properly exhibited. What physician would advise his patient to go to Newport or Long Branch, for relief from pronounced *uremia?* Or who would recommend a few forced sniffs of common air per day for *diabetes.*

Admitting all that your correspondent claimed in his lame analysis, how many chronic invalids in the ordinary practice of any physician can heed his advice to take to sawbuck and sea-side.!

On the whole, I cannot resist the conclusion that Dr. McElroy labored to no purpose, since in his concluding paragraphs, he admits that the treatment is an excellent substitute for impossible sea-shores and disagreeable, if not equally impossible, physical exercise, and by his virtual acknowledgment of its efficiency, thoroughly scientific nature, and its absolute necessity in the present state of society, he neutralizes all that he had previously said, and writes himself an ass, for having said anything.

In the Journal, for 15th May, I note Dr. Ziegler's exceptions to my reference to his labors in connection with nitrous oxide, etc. He broadly accuses me of misrepresentation and wilful injustice, since he concludes that all I know of the nature and value of protoxide of nitrogen, I derived from his publications.

I will only say that when the former article was written, I had never seen a copy of his work on the nitrous oxide, nor of any one of the papers mentioned in it as having been previously published in the various medical journals. Nearly a year ago I made diligent inquiry for his work but could not find a copy in this city—was informed that it was out of print; and not until to-day (June 1st), have I been fortunate enough to secure a copy. I have just finished a careful perusal of it, and am frank to confess that I entirely misapprehended the character and results of his researches. What I had seen of his writings, before I wrote the article in question, was simply a few extracts quoted by Barker and others, who were treating of nitrous oxide as an anæsthetic.

The misrepresentation was, therefore, entirely unconscious and unintentional on my part. It is with pleasure that I make this professional amend. I find in his work much that is interesting and instructive, and am especially pleased to learn that some one before me has asserted the office of oxygenating agents, in connection with processes of vital reparation and supply. Nearly all physiologists are ready to admit that oxygen in any form may be made an eliminator, depurent, disintegrent, resolvent, sorbefacient, etc.; very few yet believe that it may be made a *materia alimentaria* as well as a *materia medica*, and, therefore, a direct invigorator of the whole vital organism. While very few question its power as a stimulant, still fewer have learned, that in this respect, it is wholly unlike all other stimulants, in that it is not followed by corresponding depression.

I trust Dr. Ziegler will see fit to supply the profession, ere long, with a new edition of his very interesting and important work.

